# Functional VEGFA knockdown with artificial 3′-tailed mirtrons defined by 5′ splice site and branch point

**DOI:** 10.1093/nar/gkv617

**Published:** 2015-06-18

**Authors:** Kian Hong Kock, Kiat Whye Kong, Shawn Hoon, Yiqi Seow

**Affiliations:** 1 Molecular Engineering Laboratory, Biomedical Medical Sciences Institutes, 61 Biopolis Drive Proteos #03-13 Singapore 138673; 2 School of Biological Sciences, Nanyang Technological University, 60 Nanyang Drive, Singapore 637551

## Abstract

Mirtrons are introns that form pre-miRNA hairpins after splicing to produce RNA interference (RNAi) effectors distinct from Drosha-dependent intronic miRNAs, and will be especially useful for co-delivery of coding genes and RNAi. A specific family of mirtrons – 3′-tailed mirtrons – has hairpins precisely defined on the 5′ end by the 5′ splice site and 3′ end by the branch point. Here, we present design principles for artificial 3′-tailed mirtrons and demonstrate, for the first time, efficient gene knockdown with tailed mirtrons within eGFP coding region. These artificial tailed mirtrons, unlike canonical mirtrons, have very few sequence design restrictions. Tailed mirtrons targeted against VEGFA mRNA, the misregulation of which is causative of several disorders including cancer, achieved significant levels of gene knockdown. Tailed mirtron-mediated knockdown was further shown to be splicing-dependent, and at least as effective as equivalent artificial intronic miRNAs, with the added advantage of very defined cleavage sites for generation of mature miRNA guide strands. Further development and exploitation of this unique mirtron biogenesis pathway for therapeutic RNAi coupled into protein-expressing genes can potentially enable the development of precisely controlled combinatorial gene therapy.

## INTRODUCTION

The therapeutic potential of the RNA interference (RNAi) pathway, in which short double-stranded RNA mediate translational repression or degradation of targeted mRNAs, has been explored extensively with exogenous mimics such as small interfering RNAs (siRNAs) (1), short-hairpin RNAs (shRNAs) (2,3) or artificial miRNAs (amiRNAs) (4). Recently, mirtrons have been shown to be useful for gene silencing (5,6), especially when co-delivery of therapeutic genes is required. Mirtrons, first discovered in *Drosophila* (7), are natural pre-microRNA (pre-miRNA) hairpins produced as introns from mRNA splicing; they are processed by the cellular splicing machinery and lariat debranching enzyme (8) before merging with the canonical miRNA pathway during nuclear export, and are subsequently processed by Dicer-1.

**Figure 1.**
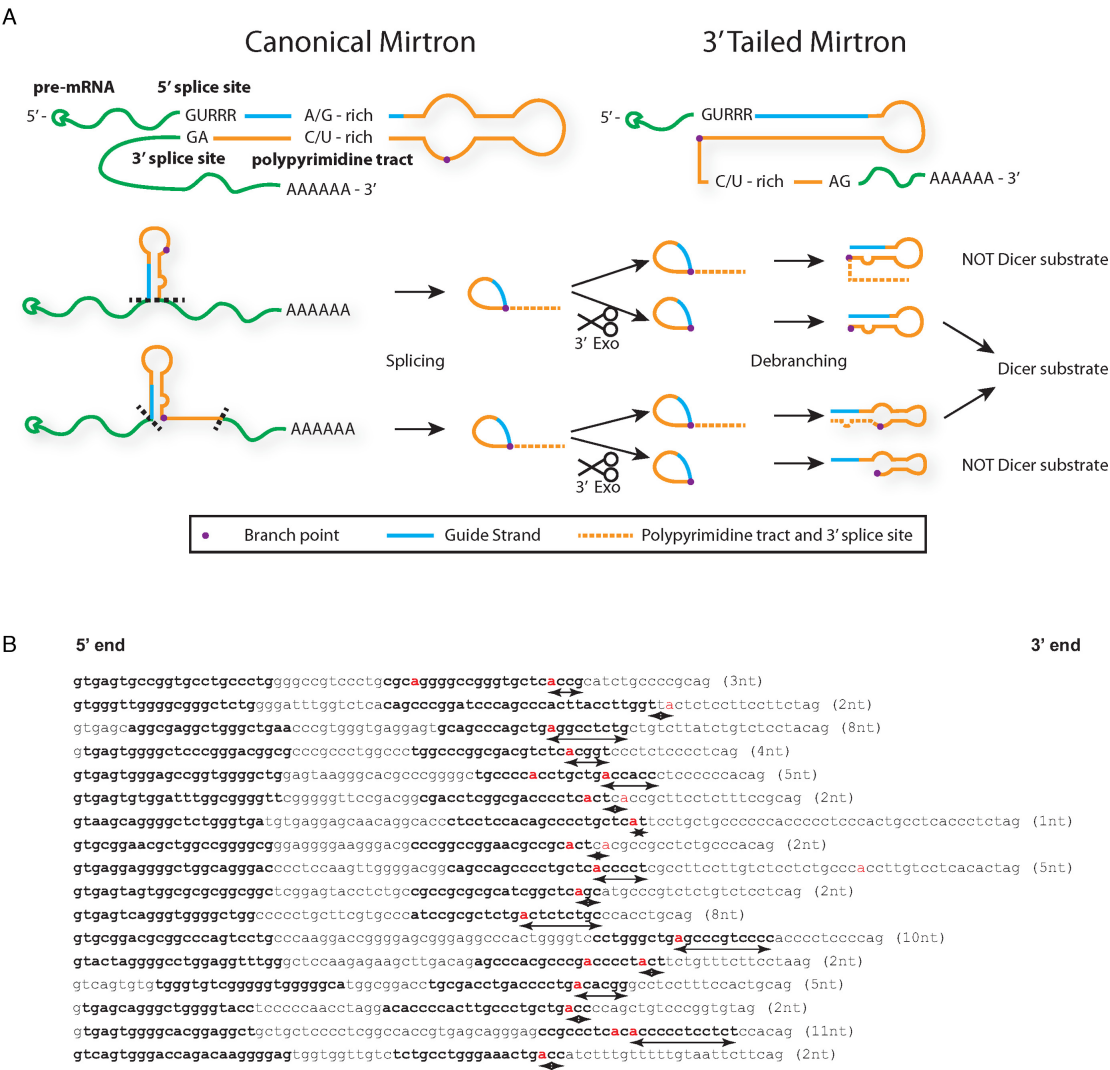
Proposed pathways for biogenesis of mirtrons and tailed mirtrons. (**A**) Canonical mirtrons have strong sequence constraints due to the need to incorporate splicing motifs within the hairpin sequence for spliceosome recognition, including the 5′ splice site (GURRR), the purine-rich 5′ arm (A/G-rich) to complement the polypyrimidine tract (C/U-rich), while tailed mirtrons are only constrained by the 5′ splice site sequence. The sequence of events following splicing is still not clear currently; thus, the interplay between 3′ exonuclease degradation by the exosome (dotted line represents sequence that is degraded) and debranching can result in different hairpins that may or may not be Dicer substrates. (**B**) Identified human 3′-tailed mirtrons from reference 13 (http://ericlailab.com/mammalian_mirtrons/hg19/). Only genuine 3′-tailed mirtrons with a 5′ arm less than 10 nt from the 5′ splice site (to exclude intronic miRNAs) and a branch point (red) that can be predicted by a branch point prediction algorithm (14) were included. Bold letters represent annotated guide and passenger strands from reference 13. Distance from the branch point to the 3′ end of the hairpin indicated in parentheses and in arrows under the sequence.

Unlike promoter-less siRNAs and RNA polymerase III-driven shRNAs, synthetic mirtrons can be controlled with an endogenous RNA polymerase II promoter, offering spatiotemporal control. However, canonical mirtrons suffer from sequence constraints due to splicing requirements that limit targetable sequences. There is a subclass of mirtrons with a 3′ tail that is particularly remarkable because most of the sequences required for splicing, specifically the polypyrimidine tract and the branch point, are located outside of the hairpin (Figure 1a) (9), thus freeing up sequence constraints (purine-rich for 5′ arm guide strands; pyrimidine-rich for 3′ arm) for synthetic mirtron design. For natural 3′-tailed mirtrons (TMirts), the precise biogenesis pathway from spliced intron to a hairpin substrate for exportin-5 and Dicer has not been elucidated, but has previously been shown to require further nucleolytic processing in flies (9). As the processing steps and sequence or structural features of TMirts are not well understood, principles behind designing artificial TMirts are not trivial to decipher. Nonetheless, successful development of artificial TMirts capable of targeting therapeutic genes of interest will have benefits for combinatorial gene therapy approaches.

Vascular endothelial growth factor A (VEGFA), which is responsible for regulating angiogenesis, presents itself as a useful therapeutic target for a variety of diseases. For example, VEGFA promotes angiogenesis and, consequently, nourishment and proliferation of tumor masses. Inhibitors of VEGFA-mediated angiogenesis are already in the clinic for cancer treatments (reviewed in (10)). The development of VEGFA TMirts unlocks future development of multi-modal gene therapy methods: the co-delivery of VEGFA TMirts together with another therapeutic gene such as a pro-drug convertase could enable targeting of tumors via multiple pathways.

In this study, we report the development of artificial 3′-tailed mirtrons (TMirts) that can be designed using widely available shRNA design algorithms with just one constraint – that of a 5′ splice site consensus sequence – unlike canonical mirtrons, which have further sequence constraints. TMirts targeting firefly luciferase and human VEGFA displayed significant knockdown when targeted to the coding sequence. TMirts were shown to be dependent on splicing. Furthermore, when compared to intronic pri-miRNA hairpins with the same guide strands, high throughput sequencing of small RNA species suggested TMirts produce a more homogenous distribution of mature RNAi effectors, a strong adherence to designed cleavage sites and more mature RNAi effectors, all of which lead to stronger knockdown. This study is the first documented use of artificial 3′ tailed mirtrons for RNAi and demonstrates that this novel technology has the potential to be exploited therapeutically.

## MATERIALS AND METHODS

### Materials

Unless otherwise stated, all chemicals were obtained from Sigma–Aldrich (Singapore) and all enzymes used were obtained from New England Biolabs (New England, MA, USA).

### Plasmids

pEGFP-Mirt, cloning of mirtrons and peGFP-NAD were previously described (11). Briefly, a BbsI-excised sequence was placed between two fragments of eGFP; introns were introduced in that site using annealed oligonucleotides, the general design shown in Supplementary Table S1 while the sequences for the individual tailed mirtrons can be found in the appropriate figures in the manuscript. Luciferase targets for Mirt-877, Mirt-1224, DMPKMirt5 and DMPKMirt13 were described previously (5,11) and the sequences for the Vegfa targets can be found in Supplementary Table S1. Briefly, 21 nucleotide sequences derived from the target mRNAs deemed to feasible targets were concatamerized and cloned into the 3′ UTR of *Renilla* luciferase by annealing complementary oligonucleotides and ligating into psiCheck2.2 (kind gift from Dr Marc Weinberg) between XhoI and NotI sites as previously described (11). The relevant shRNAs used for comparison were produced by PCR of the U6 promoter with the reverse complement of the shRNA followed by a reverse U6 primer (Supplementary Table S1). peGFP-XPO5 was described previously (5).

### Cell culture, transfection, ELISA and qRT-PCR

HEK293 cells (ATCC, Manassas, VA, USA) were cultured in Dulbecco's modified Eagle's medium (DMEM) glutamax supplemented with 10% fetal bovine serum (FBS) and antibiotics and incubated at 37°C in 5% CO_2_. Transfection of HEK293 cells were carried out with using Lipofectamine 2000 (Life Technologies, Singapore) in HEK293 cells as per manufacturer's instruction in 24-well plates. When the experiment required a control NAD plasmid or shRNA, a molar amount corresponding to the mirtron plasmid was used. The cells were imaged and lysed 48 h post-transfection. To induce hypoxia in HEK293 cells, cells were transfected with 500 ng of mirtron plasmids in 24-well plates. Twenty four hours after transfection, culture medium was changed to medium incubated overnight in the hypoxic chamber (5% CO_2_, 1% O_2_, depleted via addition of nitrogen). Cells were cultured in hypoxic conditions for 24 h before collection of cell culture media for human VEGFA Quantikine ELISA (R&D Systems, Minneapolis, MN, USA) as per manufacturer's instructions, and qRT-PCR using Trizol (Life Technologies) extraction, M-MLV reverse transcriptase (Promega) and Maxima SYBR Gold qPCR mix (Thermo Scientific, Singapore) as per manufacturers’ instructions.

### Fluorescence quantification and dual luciferase assay

The luciferase assay was performed using the Dual-Luciferase^®^ Reporter Assay System (Promega) as per manufacturer's instruction. Briefly, HEK293 cells were lysed in 100 μl of passive lysis buffer and background-subtracted eGFP fluorescence was measured in a 96-well format with 15 μl of each sample using a microplate reader (Tecan, Switzerland). Fifteen microliters of Luciferase Buffer II was added to the sample and luminescence was measured. Fifteen microliters of Stop & Glo Buffer was then added to measure *Renilla* luciferase activity. The *Renilla* luciferase signal was then normalized to the firefly luciferase signal for all luciferase experiments except for experiments in which firefly luciferase knockdown was measured, in which case, the normalization was reversed.

### High-throughput sequencing

HEK293 cells were grown in 24-well plates to 80% confluence and transfected with 500 ng of each mirtron plasmid per well. Small RNA libraries were prepared with the ‘Small RNA v1.5 Sample Prep Kit’ following the manufacturer's instructions (Illumina). Briefly, total RNA was isolated from each transfection by Trizol extraction and pooled. The RNA was ligated with a barcoded 3′ RNA adaptor modified to target small RNAs with 3′ hydroxyl groups, and then with a 5′ RNA adaptor. Reverse transcription followed by polymerase chain reaction (PCR) was performed to select for adapter ligated fragments and double-stranded DNA libraries were size selected by PAGE purification (6% TBE PAGE). Libraries were sequenced on a Genome Analyzer IIx for 50 cycles following manufacturer's protocols. The image analysis and base calling were done using Illumina's GA Pipeline. Adapters were trimmed with the Biopieces remove_adapter script and remaining sequences were aligned against full-length mirtron hairpins.

### Statistics

All experiments, unless otherwise stated, were performed in triplicates. All error bars used in this report are standard deviations. Statistical significance for luciferase knockdown was determined by two-tailed Student's *t*-test without assuming equal variance. Statistical tests were not performed for eGFP fluorescence as the fluorescence was used to answer whether a construct spliced at all, not to establish the differences between the relative levels of splicing between the constructs.

## RESULTS

### Human 3′-tailed mirtron hairpins are defined by 5′ splice site and branch point

Flynt *et al*. had suggested that *Drosophila* 3′-tailed mirtrons are debranched before RNA exosome trimming, as the RNA exosome was capable of degrading an intron down to the hairpin structure in a cell-free reaction (9). If that was the case, then there should be no correlation between branch point placement and the 3′ end of the hairpin because the exonucleolytic activity can degrade down to the hairpin after debranching. Alternatively, the mirtron can be degraded to the branch point prior to debranching and upon debranching, form the hairpin. In this case, there should be correlation between the branch point location and the 3′ end of the hairpin.

For that to happen, the order of the RNA exosome-trimming and debranching steps need to be interchangeable or concurrent within the cell (Figure 1). The 3′–5′ exonucleolytic acitivy of the yeast RNA exosome cannot digest the lariat structure as RNA lariats accumulate in yeast strains lacking debranching activity (12). If the predicted 3′ end of the hairpin structure is located away from the branch point, RNA exosome trimming prior to or concurrent with debranching may lead to 3′ ends that are either too short or too long, preventing pre-miRNA hairpin formation and probably lead to ineffective production of mature miRNA (Figure 1a).

Using a human mirtron database (13), TMirts were extracted, their mature strands identified and branch points predicted using a branch point prediction algorithm (14) (Figure 1b). The majority of human TMirts have 3′ ends within five nucleotides of the predicted branch point (13/17), suggesting human TMirts are preferentially defined by their branch points after exonucleolytic digest.

### Defined branch point at base of hairpin and a strong polypyrimidine tract produces effective artificial 3′-tailed mirtrons

**Figure 2.**
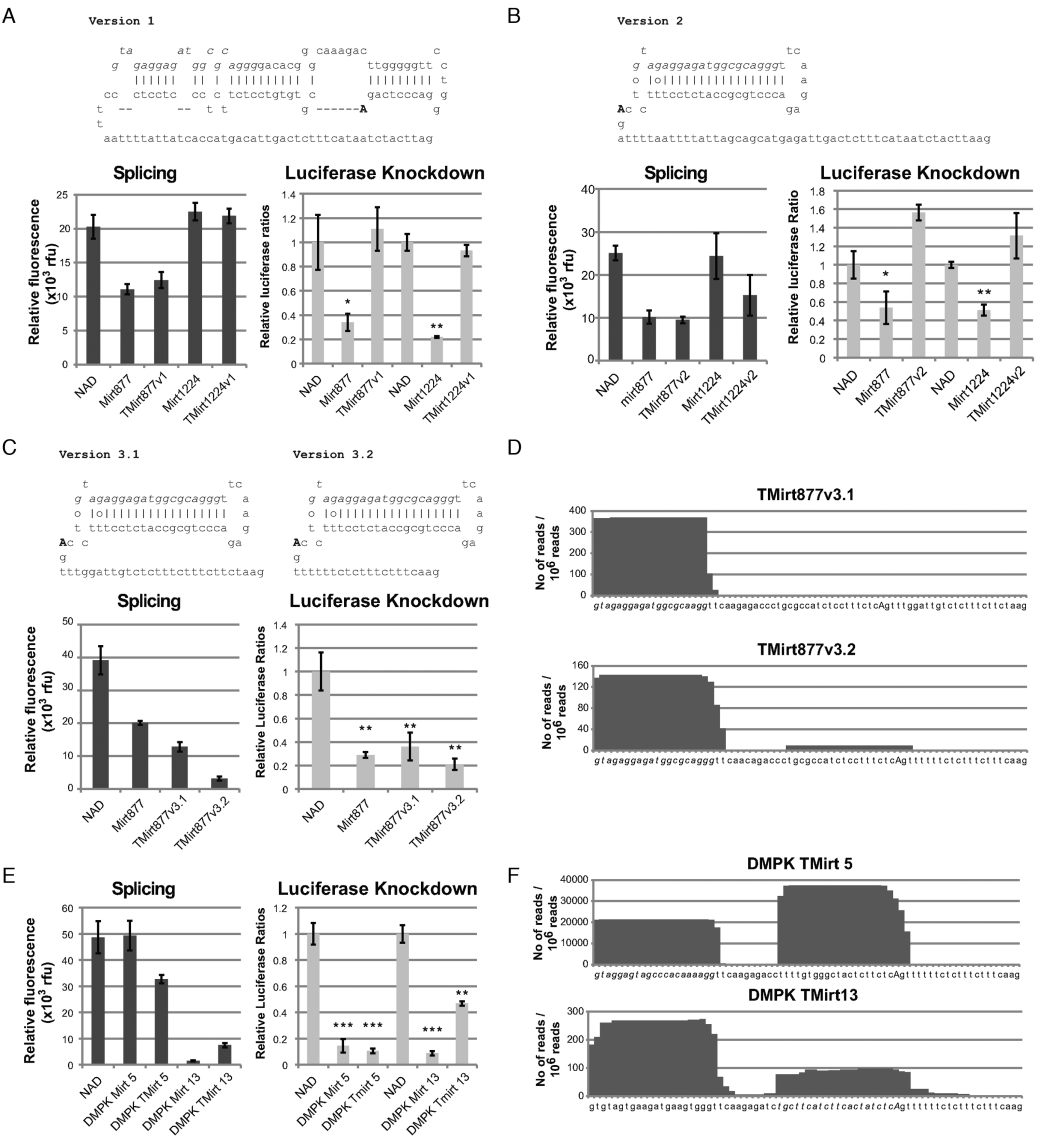
Development of effective 3′-tailed mirtrons. HEK cells were transfected with 100 ng of psiCheck2.2 targets, with target sequences in the 3′ UTR of *Renilla* luciferase, and 500 ng of mirtron plasmid, and lysed two days later for fluorescence measurement and luciferase activity. (**A–C**) eGFP fluorescence quantification, *Renilla* luciferase knockdown and different TMirt designs with hsa-mir-877 and mmu-mir-1224 guide strands in a pEGFP-Mirt vector. Guide strands and putative branch points are marked by italic and uppercase text respectively. (**D**) High-throughput sequencing was performed with 18–25nt RNA from HEK293 cells transfected with TMirt877v3.1 and v3.2. Sequences that were aligned to mirtron sequences were collected, 3′ tail removed and aligned to chart the frequency of individual nucleotides appearing in the small RNA species harvested from the cells. Nucleotides in italics denote guide strand, uppercase denotes branch point. (**E**) TMirts based on canonical mirtrons designed against DMPK were compared to the parent mirtrons based on splicing efficiency (eGFP fluorescence) and *Renilla* luciferase knockdown. (**F**) High-throughput sequencing of small RNAs from HEK293 cells transfected with 500 ng of the relevant plasmids. *P < 0.05, **P < 0.01, ***P < 0.001 versus NAD control, *n* = 3 biological replicates for each experiment. Error bars reflect standard deviation.

Given the rules governing natural 3′-tailed mirtrons, we attempted to generate artificial TMirts nested within eGFP that could target transcripts of interest. The expression of eGFP will be a guide to the presence of splicing. The relative levels of eGFP can be influenced by other factors like transcription efficiency mediated by the presence of an intronic sequence, thus eGFP fluorescence is not meant to be used as a tool to compare relative levels of splicing. As we did not want to contend with the potency of the guide strand, we based the initial designs on mature hsa-miR-877 and mmu-miR-1224 (Supplementary Figure S1, derivative designs for miR-877 in Figure 2 and miR-1224 in Supplementary Figure S1) – canonical mirtrons with the guide strands in the 5′ arm previously shown to be functional against reporter targets located in the 3′ untranslated region (3′ UTR) of *Renilla* luciferase (11). We first replaced the 3′ splice site of the natural mirtron hairpins with a longer polypyrimidine tract and the 3′ splice site of intron 6 of human NDUFS1, an intron that was previously used as a negative control for mirtron experiments (NAD) (11). Splicing would still depend on the predicted branch point located within the hairpin, located at approximately the same distance from the 3′ splice site as the predicted branch point in NDUFS1 intron 6 (Figure 2a, v1). Despite successful splicing based on eGFP fluorescence levels, no luciferase knockdown was detectable with these constructs in HEK293 cells. Unsurprisingly, the lack of knockdown may be because the branch point is located too far away from the 3′ end of the hairpin, thus resulting in the degradation of the 3′ arm of the hairpin prior to debranching or alternatively, the spliceosome preferentially utilizes adenines closer to the 3′ splice site, resulting in long 3′ tailed hairpins that are not functional shRNAs.

In an attempt to improve the TMirt design, instead of the original hairpin, a minimal hairpin with a highly complementary 3′ arm was used to remove both the polypyrimidine tract and the branch point from the hairpin sequence. This design also frees up sequence constraints on the guide strand that plague the development of artificial mirtrons. The branch point and polypyrimidine tract of NDUFS1 intron 6 were inserted 3′ of the hairpin (v2). Both the miR-877-derived and miR-1224-derived tailed mirtron spliced relatively well but, unfortunately, no knockdown was observed with this design (Figure 2b).

As the polypyrimidine tract of NDUFS1 intron 6 is rather long and its branch point ill-defined, ideal polypyrimidine tracts of different lengths were substituted into the TMirt design; adenine nucleotides were excluded from the polypyrimidine sequence to prevent the introduction of potential branch points. The third iterations of tailed mirtron designs (v3.1 and v3.2) with the miR-877 guide strand were spliced and resulted in knockdown of the luciferase target comparable to the parental mir-877 mirtron (Figure 2c). High-throughput sequencing of small RNAs produced in HEK293 cells transfected with these constructs demonstrated that the guide strand produced by the tailed mirtron is as designed, with a very precisely defined 5′ end (Figure 2d). Surprisingly, shortening the stem loop to a short shRNA loop and increasing the complementarity of the duplex has resulted in stronger bias toward the 5′ arm of the duplex compared to the canonical miR-877 placed in the same context (11). Although both designs spliced and can be used to design TMirts, we chose to design subsequence TMirts with the v3.2 backbone as the v3.2 design resulted in stronger luciferase knockdown and has a shorter tail.

Next, to demonstrate that this design was generally applicable, artificial mirtrons previously designed against DMPK (5) were converted to TMirts (Supplementary Figure S2). DMPK TMirt 5 and DMPK TMirt 13 were designed with the guide strand in the 5′ arm and 3′ arm, respectively. These constructs were similarly able to induce knockdown of their respective luciferase reporter targets at levels comparable to their parent mirtrons (Figure 2e). More importantly, sequencing of the small RNAs produced validates the TMirt design and demonstrates precision of TMirt processing (Figure 2f). However, the large number of passenger strand reads from DMPK TMirt 5 which may result in off-target effects means that there is still room for improvement of the design.

### 3′-Tailed mirtrons can effect gene knockdown when targeted to coding regions

Canonical mirtrons effective against luciferase 3′ UTR targets are relatively ineffective when the same target sequences are in the coding region, which may prove to be a hindrance to widespread application. This may be due to the low levels of mature species being generated for synthetic canonical mirtrons, which we have so far been unable to detect with northern blots (unpublished observations). This is in contrast to other groups that are able to detect generation of mature miRNAs via Northern blots of natural canonical mirtrons in their original exonic context (7,15). Thus we sought to investigate if tailed mirtrons would fare better.

**Figure 3.**
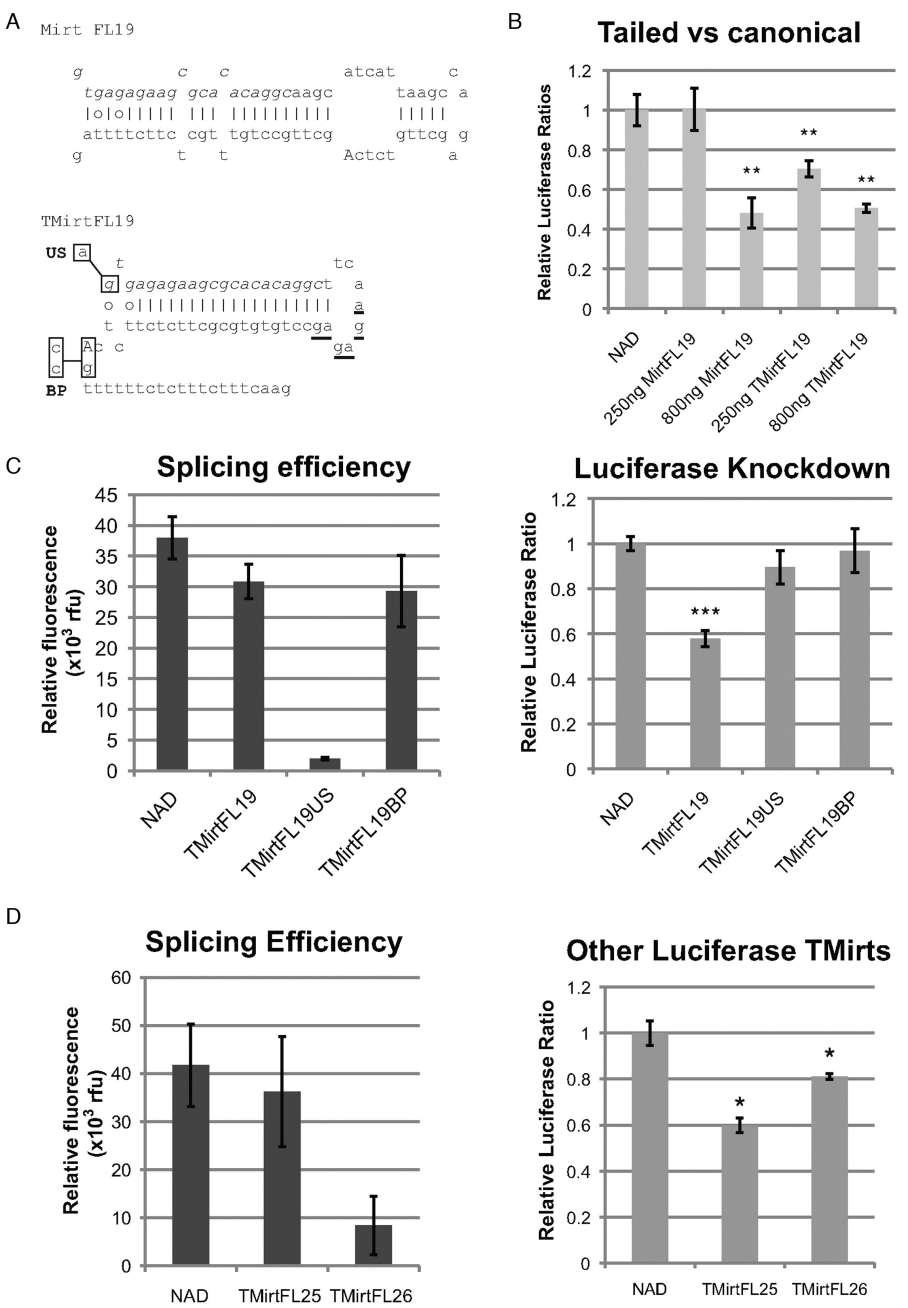
3′-Tailed mirtrons effect strong knockdown when targeted to the coding region. (**A**) Sequence of TMirtFL19 compared to MirtFL19. Mutations made to the 5′ splice site (US) from GUGAG to AUGAG and branch point (BP) from CTCAG to CTCCC are indicated by substitutions of the nucleotides within the boxes. Nucleotides in italics denote guide strand, uppercase denotes branch point. (**B**) HEK293 cells were transfected with 100 ng of psiCheck2.2 and 0, 250 or 800 ng of peGFP-MirtFL19 or peGFP-TMirtFL19, and topped up to 900 ng of nucleic acids with peGFP-NAD. Cells were harvested two days after transfection. (**C**) HEK293 cells were transfected with 100 ng of psiCheck2.2, and 500 ng of mirtrons/NAD. Cells were harvested two days after transfection. (**D**) HEK293 cells were transfected with 100 ng of psiCheck2.2, and 500 ng of mirtrons / NAD. Cells were harvested 2 days after transfection. *P < 0.05, **P < 0.01, ***P < 0.001 versus NAD control, *n* = 3 biological replicates for each experiment unless otherwise stated. Error bars reflect standard deviation.

Artificial mirtrons targeted to the firefly luciferase coding region using the mirtron design algorithm were previously found to be unable to result in strong knockdown despite corresponding shRNAs utilizing the same guide strands achieving strong knockdown (16). Hence, in spite of the guide strand having strong knockdown potential, canonical mirtrons might have produced too few mature guide strands to effect knockdown. In particular, FL19 in the context of an shRNA demonstrated strong knockdown, while mediocre knockdown was achieved in the corresponding mirtron. Converting FL19 to a TMirt design resulted in a dose-dependent knockdown of Firefly luciferase (Figure 3a and b). This demonstrates that TMirts can target coding regions of genes, in addition to 3′ UTR targets. Knockdown with 250 ng of TMirtFL19 was notably stronger than knockdown with 250 ng of MirtFL19 despite reaching saturation at 800ng, suggesting that the TMirt designs may be more effective than the canonical design at targeting ORFs.

### TMirt-mediated knockdown is dependent on splicing

Mutation of the guanine nucleotide of the 5′ splice site of TMirtFL19 to adenine (US) abrogated splicing based on eGFP fluorescence and knockdown (Figure 3c). Mutation of the branch point AG to pyrimidines CC (BP) elongates the polypyrimidine tract and probably shifts the predicted branch point further up the hairpin in the 5′ direction to one of the triple AG dinucleotides (underlined in Figure 3a) in the hairpin loop which are predicted to be the new branch point by the branch point prediction algorithm, resulting in strong splicing but absolutely no knockdown. These mutants demonstrate that knockdown is dependent on precise splicing to generate the pre-miRNA hairpin.

New TMirts targeting opening reading frames (ORFs) in firefly luciferase, with the guide strand in both 5′ arm and 3′ arm of the hairpin, were also designed with a modified mirtron target identification algorithm without the preference for polypyrimidine or polypurine tracts within the hairpin sequence (Supplementary Figure S3). These also resulted in significant knockdown of Firefly luciferase (Figure 3d), validating the TMirts as an effective means to target protein-coding regions of genes.

### TMirts against VEGFA results in functional knockdown of VEGFA levels

**Figure 4.**
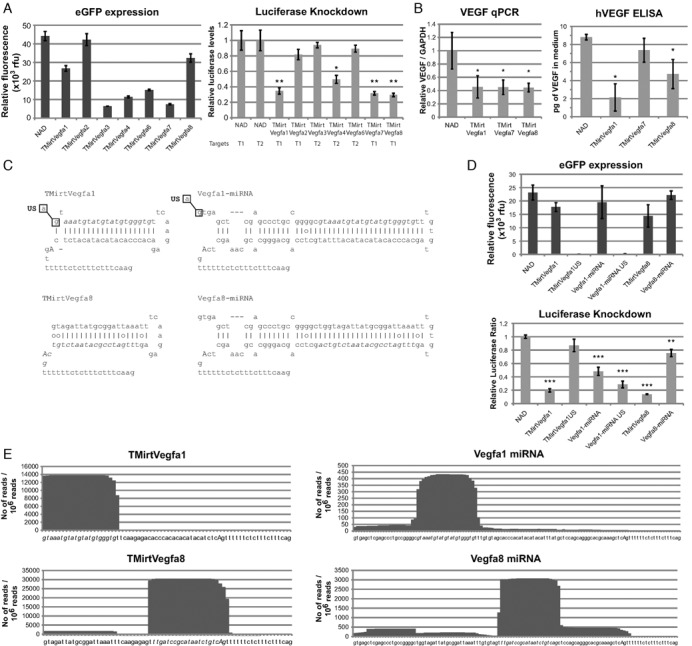
3′-Tailed mirtrons targeted against VEGFA result in functional knockdown of VEGF-A in a cell culture model of hypoxia. (**A**) HEK293 cells were transfected with 500 ng of NAD/TMirts and 100 ng of the matched psiCheck2.2 targets (T1 and T2 contain concatamers of the target sequences in the 3′ UTR). Cells were harvested two days after transfection. (**B**) HEK293 cells were transfected with 500 ng of mirtrons/NAD and subjected to hypoxic conditions 1 day after transfection for 24 h before harvesting. qRT-PCR of VEGF was performed using RNA harvested from the cells and normalized against GAPDH. ELISA was performed on the cell culture supernatant; total human VEGF secreted in 500 μl of media, as measured by ELISA, is indicated. (**C**) Sequences of TMirts compared to their miRNA equivalents. Mutations made are indicated by substitutions of the nucleotides within the boxes. (**D**) HEK293 cells were transfected with 500 ng of NAD/TMirts mutants / miRNA mimics and 100 ng of the matched psiCheck2.2 VEGFA T1. Cells were harvested two days after transfection. (**E**) High-throughput sequencing of small RNAs from HEK293 cells transfected with 500 ng of the relevant plasmids. *P < 0.05, **P < 0.01, ***P < 0.001 versus NAD control, *n* = 3 biological replicates for each experiment unless otherwise stated. Error bars reflect standard deviation.

Some tumor cells secrete VEGFA to promote angiogenesis. Efforts to target VEGFA signaling with siRNA (17) and monoclonal antibodies (18) have gained clinical traction, although one can only be cautiously optimistic due to the weak effect on tumor cell survival. The ability to target tumors concurrently from multiple angles may help improve the efficacy of therapeutics. VEGFA mirtrons can be one option for co-delivery of a suicide gene with RNAi modalities. Thus, we designed TMirts targeted against VEGFA and first tested for knockdown using luciferase reporter constructs with the target sequences cloned as concatamers in the 3′ UTR of *Renilla* luciferase. Designs 1, 2, 3 and 6 targeted the 3′ UTR of VEGFA and the rest targeted the coding region. All mirtrons spliced to different extents, but only designs 1, 4, 7 and 8 achieved luciferase knockdown (Figure 4a, sequences in Figure 4c and Supplementary Figure S4). Of the four constructs, three (designs 1, 7 and 8) were chosen for further validation studies in a functional assay using a hypoxia-induced VEGF expression model. HEK293 cells were transfected with the TMirts and incubated in a hypoxic chamber for 24 h. Subsequently, cell culture media was collected and RNA harvested for quantitative RT-PCR. qRT-PCR results suggest that endogenous VEGFA mRNA was degraded by the mirtrons; subsequently, VEGFA secretion was also strongly inhibited by TMirtVegfa1 and less so by TMirtVegfa8 (Figure 4b). These results demonstrate that TMirts against VEGFA can potently inhibit endogenous VEGFA secretion and thus can be a potential component in cancer gene therapeutics.

### VEGFA TMirts are more precisely processed than miRNA mimics designed with the same guide strand

To evaluate the differences between intronic miRNA mimics and TMirts, guide strands for VEGFA-TMirt 1 and 8 were incorporated into intronic miRNAs mimics based on miR-106b cluster as previously described (19) (Figure 4c) and these miRNA mimics were capable of effecting knockdown albeit at a slightly lower level compared to tailed mirtrons (Figure 4d). The difference in biogenesis was highlighted by the mutation that abrogated the 5′ splice site in the tailed mirtron and miRNA mimic (US) and prevented proper splicing of the eGFP reporter (Figure 4d). The mutation resulted in the loss of TMirtVegfA1-mediated knockdown but not of the corresponding miRNA-mimic, suggesting that splicing is required to generate functional mature TMirts but not miRNA mimics. Interestingly, inhibition of splicing for Vegfa miRNA-mimic 1 seemed to increase the knockdown, possibly because lariat formation and subsequent degradation could compete with miRNA biogenesis. It is known that Drosha processing of pri-miRNA can result in heterogenous ends (20) and thus can result in different mature miRNA species being produced. Splicing, on the other hand, is very precise out of necessity, thus we wanted to establish if TMirts produced a more homogenous population of mature miRNA species. High throughput sequence of small RNA species (Figure 4e; Supplementary Figure S5) demonstrated that TMirts produced a largely homogenous population of mature miRNA species very close to the designed guide strand sequence while the miRNA mimics were much more heterogenous and also yielded other unintended species that may resulted in off target effects. Furthermore, the number of reads per million small RNA sequences suggests that greater numbers of mature miRNA species were being produced by TMirts than miRNA mimics. A caveat to this observation is that the intronic miRNA-mimics designs were not tested against all TMirt designs so this may not be true with all miRNA-mimics. The increase in number of mature species and the precision of biogenesis resulted in greater knockdown using the luciferase reporter. This result suggests that although TMirts have more design constraints that intronic miRNA-mimics, they may be more advantageous than intronic miRNA mimics for applications requiring co-delivery of RNAi and protein-coding sequences.

### Design features for TMirts

Based on small RNA sequencing of the TMirts, a few design principles to produce precise mature guide strands can be elucidated. The key features of the design, as exemplified with TMirtVegfa1and TMirtVegfa8 (Figure 4c) include (i) a canonical GU 5′ splice site followed by three purines, although splicing may still occur if one or two of the purines was substituted with pyrimidines, (ii) a short hairpin loop to connect the 5′ arm guide / passenger strand to the 3′ arm passenger/guide strand, (iii) designing the hairpin to end one nucleotide after the branch point adenine nucleotide and (iv) a strong branch point and polypyrimidine tract.

## DISCUSSION

In this study, we demonstrate synthetic TMirts designs capable of mediating slicing-dependent gene knockdown for a variety of targets, most notably reducing VEGF protein levels in a hypoxia-induced model. We further show that TMirts can be more efficacious in gene knockdown, compared to miRNA mimics and canonical mirtrons that utilize the same guide strand. TMirts can be designed for a range of transcripts based on several design principles. These developments in synthetic TMirts hence present new possibilities for targeted gene knockdown, with numerous advantages that can be harnessed for therapeutic interventions. The field of RNAi-based therapeutics is an intensive area of research, with numerous therapeutic shRNAs and siRNAs advanced through various stages of clinical trials. Naked siRNA therapeutics have been utilized in anti-VEGF clinical trials for age-related macular edema and diabetic macular edema (21). However, these therapeutics face numerous challenges – naked siRNA molecules are susceptible to degradation, and RNAi-based therapeutics face challenges in terms of knockdown efficacy, miRNA-specificity and also potential off-target effects related to the sequences of both the miRNA guide strand and passenger strand (21).

TMirts offer various advantages over alternative forms of RNAi therapies in niche applications. TMirts are as effective in gene knockdown as intronic pre-miRNA mimics utilizing the same guide strands, but are shorter to construct and easier to incorporate. Instead of utilizing naked siRNA or constructs under the control of RNA Pol III promoters, TMirts can be delivered within a vector under the control of specific RNA Pol II promoters, allowing for spatiotemporal tissue specificity. Small RNAs are commonly expressed using Pol III promoters such as H1 and U6; however, deep sequencing of small RNAs showed that the initiation of transcription from these promoters can be variable and sequence context-dependent (22). The variability of expressed transcripts can result in potentially less efficacious gene knockdown and/or increased off-target activity. Although adhering to particular design guidelines can increase initiation accuracy, this comes at the cost of increased sequence constraints. In addition, these H1 and U6 promoters are highly robust and constitutive in numerous cell types: they can drive high levels of expression of small RNAs, potentially inducing toxicity and tissue damage through the saturation of the endogenous miRNA pathway (23,24).

As the generation of pre-miRNA constructs from TMirts is absolutely dependent on splicing, the conservation of the splice donor and branch point sites allows for well-defined transcript sequences with low sequence variability at the 5′- and 3′-ends in contrast to the variability associated with Pol III promoter-driven expression and Drosha processing. The development of TMirts offers a distinct advantage over canonical mirtrons by removing most splicing sequence constraints inherent in mirtron hairpin design. In TMirts, the 3′-tail of mirtrons accommodates the polypyrimidine tract and 3′ splice acceptor site, relieving the sequence constraints on the hairpin. Furthermore, our results show that although the splicing efficiency of a tailed mirtron construct may be lower than that of a comparable mirtron construct, both constructs may still demonstrate comparable gene knockdown efficacies (Figure 2c). This suggests that the tailed mirtron construct may be a more suitable substrate in the production of mature miRNA species than canonical mirtron constructs.

Although the TMirt hairpin is defined by splicing, generation of the mature double stranded miRNA precursor is via Dicer, which is known to produce heterogenous ends (25,26). Indeed, our sequencing data does suggest some heterogeneity in the ends processed by Dicer, which may result in off-target effects. Fortunately, Gu *et al*. has demonstrated with U6 promoter-generated shRNA hairpins that shortening the hairpin such that the 5′ arm ends exactly at the loop results in a very high degree of homogeneity after Dicer processing (26). Although not implemented within this study, this design can be easily implemented in current TMirt designs to precisely define both ends of the mature RNAi species.

It is notable that there is no correlation between splicing efficiency as measured with eGFP fluorescence and knockdown. This is not particularly surprising given that knockdown is dependent on splicing, post-splicing modifications, nuclear export, recognition and processing by Dicer, strand selection by RISC and intrinsic knockdown efficiency of the guide strand sequence, thus it is unlikely that splicing alone can explain the differences in knockdown. However, the inclusion of the TMirts does seem to have an effect on the expression of eGFP, which suggests that expression of the parental gene may be reduced by the incorporation of TMirts and this may be undesirable in certain cases. However, this issue is not unique to TMirts and should be considered whenever additional intronic sequences are added to coding regions.

The high-throughput sequencing results suggested that strand selection bias of DMPK TMirt 5 and DMPK TMirt 13 could be improved although the other TMirts sequenced had much better strand selection bias. Selection of the passenger strand rather than the guide strand can result in off-target effects and thus strand selection is an important design constraint for TMirts. Although guide and passenger selection of duplex RNAi effectors is dictated a number of different factors (27), low internal stability at the guide strand 5′ terminus (mismatches, majority A-T base pairs) seems to be a design constraint that can help bias strand selection toward the desired guide strand (28). The TMirts were designed with this principle but as shown with DMPK TMirt 5 and DMPK TMirt 13, it is not foolproof. Further insights into strand selection bias elucidated in the future may be able to improve the designs of TMirts.

Both mirtron and TMirt biogenesis depend on debranching of the spliced lariat to generate the pre-miRNA hairpin; this function is solely dependent on the lone debranching enzyme, DBR1. As debranching activity can be detected in the cytoplasm (29), lariats accumulate in the cytoplasm when DBR1 is deleted in yeast (30) and there is evidence the DBR1 shuttles between the nucleus and cytoplasm (31), there is likely an endogenous exportin-5-independent nuclear export pathway for branched lariats. Thus, it is tempting to speculate that TMirts could be exported via this pathway before being debranched in the cytoplasm, thus offering an alternative nuclear export pathway to exportin-5-mediated export. Further experiments to investigate TMirt nuclear export may help shed light on this.

To date, little is known mechanistically about the processing of TMirts and mirtrons to generate mature miRNA species, especially the involvement of the RNA exosome and DBR1 and the effects stable secondary structures play given their prevalence (32). In addition, much is still unknown about the nature of the exosome degradation substrate. The synthetic TMirts developed in this study represent constructs with fewer secondary structure and regulatory determinants compared to endogenous mirtrons, and their lower complexity may serve as a useful platform for elucidation of details of the mirtron maturation pathway and mechanisms of miRNA biogenesis from TMirts and mirtrons, independent of heterologous regulatory factors.

Intriguingly, while splicing-derived mirtron hairpins provide a major contribution to the diversity of endogenous RNA interference substrates in mammals, the utilization of the 3′-tailed mirtron pathway appears to be comparatively limited in mouse and human sequence data. However, the evolutionary sequence conservation of several 3′-tailed mirtrons appears to indicate conserved pathways for 3′-tailed mirtron maturation (33,34). Further investigation into the nature, mechanism and saturation of the 3′-TMirt maturation pathway can shed light on their biological importance.

There is much potential for the use of TMirts in combination with gene therapy methods to delivery concurrent gene knockdown and replacement, as evident in the use of TMirts in tandem with eGFP in this and other studies (5,6). Multiple TMirts may be combined in the same vector, in a manner reminiscent of dual-targeting siRNAs (21) – allowing for multiple sites on a single mRNA molecule or different mRNA molecules to be targeted – or pri-miRNA cluster (23) – in which multiple pri-miRNA mimics are present within a single transcript. This would allow for combinatorial miRNA therapeutics and contribute toward possible additive or enhanced levels of gene knockdown. In conclusion, this study demonstrates the first use of TMirts as artificial RNAi molecules that may be best suited for combinatorial gene therapy and adds a new tool in the therapeutic and diagnostic toolbox for genetic manipulation.

## SUPPLEMENTARY DATA

Supplementary Data are available at NAR Online.

SUPPLEMENTARY DATA
