# Supplementary material for: Functional VEGFA knockdown with artificial 3′-tailed mirtrons defined by 5′ splice site and branch point
Source: Nucleic Acids Res. 2015 Jun 18;43(13):6568–78. doi: 10.1093/nar/gkv617 (PMC4513878; doi:10.1093/nar/gkv617)
Supplement: SUPPLEMENTARY DATA [file supp_43_13_6568__index.html]

Functional VEGFA knockdown with artificial 3′-tailed mirtrons defined by 5′ splice site and branch point — SUPPLEMENTARY DATA 

# Functional VEGFA knockdown with artificial 3′-tailed mirtrons defined by 5′ splice site and branch point

## SUPPLEMENTARY DATA

- SUPPLEMENTARY DATA
