# Supplementary material for: Functional VEGFA knockdown with artificial 3′-tailed mirtrons defined by 5′ splice site and branch point
Source: Nucleic Acids Res. 2015 Jun 18;43(13):6568–78. doi: 10.1093/nar/gkv617 (PMC4513878; doi:10.1093/nar/gkv617)
Supplement: SUPPLEMENTARY DATA [file supp_gkv617_nar-00810-y-2015-File006.pdf]

Primer	Sequence
**pEGFP-Mirt, psiCheck2.2, shRNA and DMPK Cloning**	
**Mirtron cloning**	5’-caag – mirtron sequence forward-3’ and<br>5’-cgtc – mirtron sequence reverse-3’
**VEGFA T1<br>for (Mirt 1, 2, 7, 8)**	5’-tcgatgcagattatgcggatcaaacctgtagacacacccacccacccaca<br>tacatacatttatgcggatcaaacctcaccaa-3’
	5’-ggccttggtgaggtttgatccgcataaatgtatgtatgtgggtgggtggg<br>tgtgtctacaggtttgatccgcataatctgca-3’
**VEGFA T2<br>for (Mirt 3, 4, 5, 6)**	5’-tcgagccttgccttgctgctctacctccacatgatcttttttttgt<br>cccactaatgttattggtgtcttcac-3’
	5’-ggccgtgaagacaccaataacattagtgggacaaaaaaaagatcat<br>gtggaggtagagcagcaaggcaaggc-3’
**shRNA U6 Reverse**	5’-ggtgtttcgtcctttccacaa – *mirtron sequence reverse*-3’
**U6 Forward**	5’-ggctgcaggtcgacggat-3’
**Quantitative PCR**	
**hGAPDH F**	5’-aaggtgaaggtcggagtcaa-3’
**hGAPDH R**	5’-gaagatggtgatgggatttc-3’
**VEGFA F**	5’-cggatcaaacctcaccaaggc-3’
**VEGFA R**	5’-agggaggctccttcctcct-3’

Supplementary Table S1. Oligonucleotides used in this study.

```
Natural Mirt877

    ta      at  c c          g caaagac         c
   g  gaggag  gg g agggacacg g       ttgggggtt c
      ||||||  || | |||||||||| |       ||||||||| t
  cc  ctcctc  cc c tctcctgtgt c       gactcccag g
ga  --      --  t t          g ------A         g


Natural Mirt1224

 g      cu           ------    u   aucau      c
  ugagga  ggggaggugg      aggg agc     uagagc a
  ||||||  ||||||||||      |||| |||     ||||||
  acuccu  cuucuccacc      uccc ucg     gucucg g
 g      cu           ccccuc    -   acucu      a

TMirt1224 Version 1

 g      ct           ------    u   aucau      c
  tgagga  ggggaggtgg      aggg agc     uagagc a
  ||||||  ||||||||||      |||| |||     ||||||
  acuccu  cuucuccacc      uccc ucg     gucucg g
 t      cu           ccccuc    -   acucu      a
 t
  aatttattatcaccatgacattgactctttcataatctacttag

TMirt1224 Version 2

    t                  tc
   g gaggactggggaggtggt  a
   | o||o||||||||||||||  a
   c tccttgacccctccacca  g
Act t                  ga
g
atttaattttattagcagcatgagattgactctttcataatctacttaag
```

Supplementary Figure S1. Mirt-877 and Mirt-1224 Natural and Tailed Mirtron designs. Cyan nucleotides indicate guide strand. Red nucleotides denotes branch point.

```
DMPKTmirt5

   t                    tc
  g aggagtagcccacaaaaggt  a
  o |o||||||||||||||||||  a
  t ttctcatcgggtgttttcca  g
Ac c                    ga
g
tttttctctttctttcaag

DMPKTMirt13

   t                    tc
  g gtagtgaagatgaagtgggt  a
  o o||||||||||||||oo|o|  a
  t tatcacttctacttcgtcta  g
Ac c                    ga
g
tttttctctttctttcaag
```

Supplementary Figure S2. DMPK Tailed Mirtron designs. Cyan nucleotides indicate guide strand. Red nucleotides denotes branch point.

```
TMirtFL25

   t                    tc
  g tagacaggtcgtacttgtct  a
  o ||||||||||||||||||||  a
  t atctgtccagcatgaacaga  g
Ac c                    ga
g
tttttctctttctttcaag

TMirtFL26

                        tc
  gtgaacagcatgggcatttaat  a
  o|||||||||||||||||||||  a
  tacttgtcgtacccgtaaatta  g
Ac                      ga
g
tttttctctttctttcaag
```

Supplementary Figure S3. Firefly Luciferase Tailed Mirtron designs.Cyan nucleotides indicate guide strand. Red nucleotides denotes branch point.

```
TMirtVegfa2

   t                    tc
  g gggtgggtgtgtctacaggt  a
  o |||||||||||||||||||   a
  t cccacccacacagatgtcca  g
Ac c                    ga
g
tttttctctttctttcaag

TMirtVegfa3

   t                    tc
  g gaagacaccaataacattat  a
  o |||||||||||||||||||   a
  t cttctgtggttattgtaata  g
Ac c                    ga
g
tttttctctttctttcaag

TMirtVegfa4

   t                    tc
  g agagcagcaaggcaaggctt  a
  o |||||||||||||||||||   a
  t tctcgtcgttccgttccgaa  g
Ac c                    ga
g
tttttctctttctttcaag

TMirtVegfa6

   t                    tc
  g gggacaaaaaaaagatcatt  a
  o |||||||||||||||||||   a
  t ccctgttttttttctagtaa  g
Ac c                    ga
g
tttttctctttctttcaag

TMirtVegfa7

                        tc
  gtggatcaaacctcactaatat  a
  oo|||||||||||||o|||||   a
  tgcctagtttggagtggttata  g
Ac                      ga
g
tttttctctttctttcaag
```

Supplementary Figure S4. Human VegfA Tailed Mirtron designs. Cyan nucleotides indicate guide strand. Red nucleotides denotes branch point.

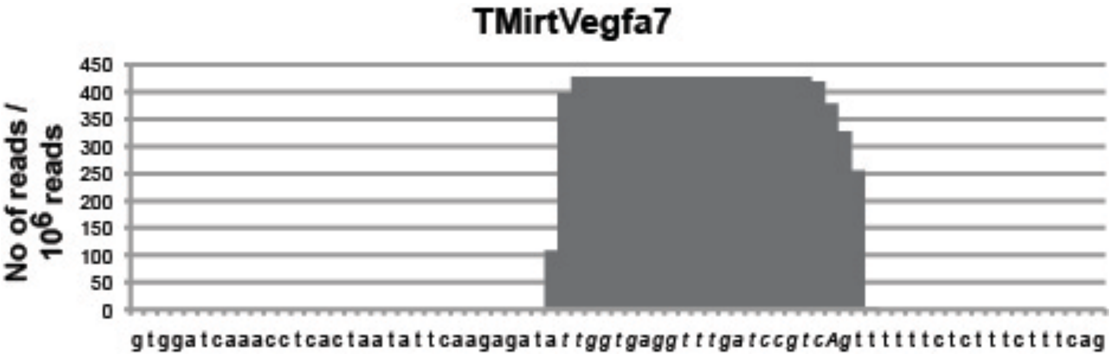

Supplementary Figure S5. High throughput sequencing of small RNA from cells transfected with 500ng peGFP-TMirtVegfa7. Nucleotides in italics indicate guide strand; capital letter denotes branch point.
